# Recent Insights on Supraspinal Astrocytes in Chronic Pain

**DOI:** 10.1007/s11064-025-04503-x

**Published:** 2025-08-23

**Authors:** Alessandro Di Spiezio, Marta Gómez-Gonzalo, Angela Chiavegato, Micaela Zonta

**Affiliations:** 1 Neuroscience Institute, National Research Council (CNR), Padova, Italy; 2 Department of Biomedical Sciences, University of Padova, Padova, Italy; 3 Padova Neuroscience Center, University of Padova, Padova, Italy

**Keywords:** Astrocytes, Nociception, Central sensitization, Chronic pain, Behaviour

## Abstract

Chronic pain is a debilitating, life-altering condition that affects a significant portion of the global population, impacting approximately one fifth of people in Europe and one third of people worldwide. Clinical and experimental efforts are increasingly converging to deepen our comprehension of the molecular, cellular and circuit-level mechanisms underlying persistent pain. While most studies have traditionally focused on alterations of nociceptive pathways in neurons, growing evidence highlights the critical role of astrocytes in modulating these pathways and contributing to the development of the central sensitization that characterizes chronic pain. Moreover, astrocytes are also implicated in pain-associated maladaptive behaviours and cognitive impairments. In this context, we review the latest findings on astrocyte involvement in chronic pain and its related mood and cognitive comorbidities.

**Graphical Abstract:**

**Figure:**
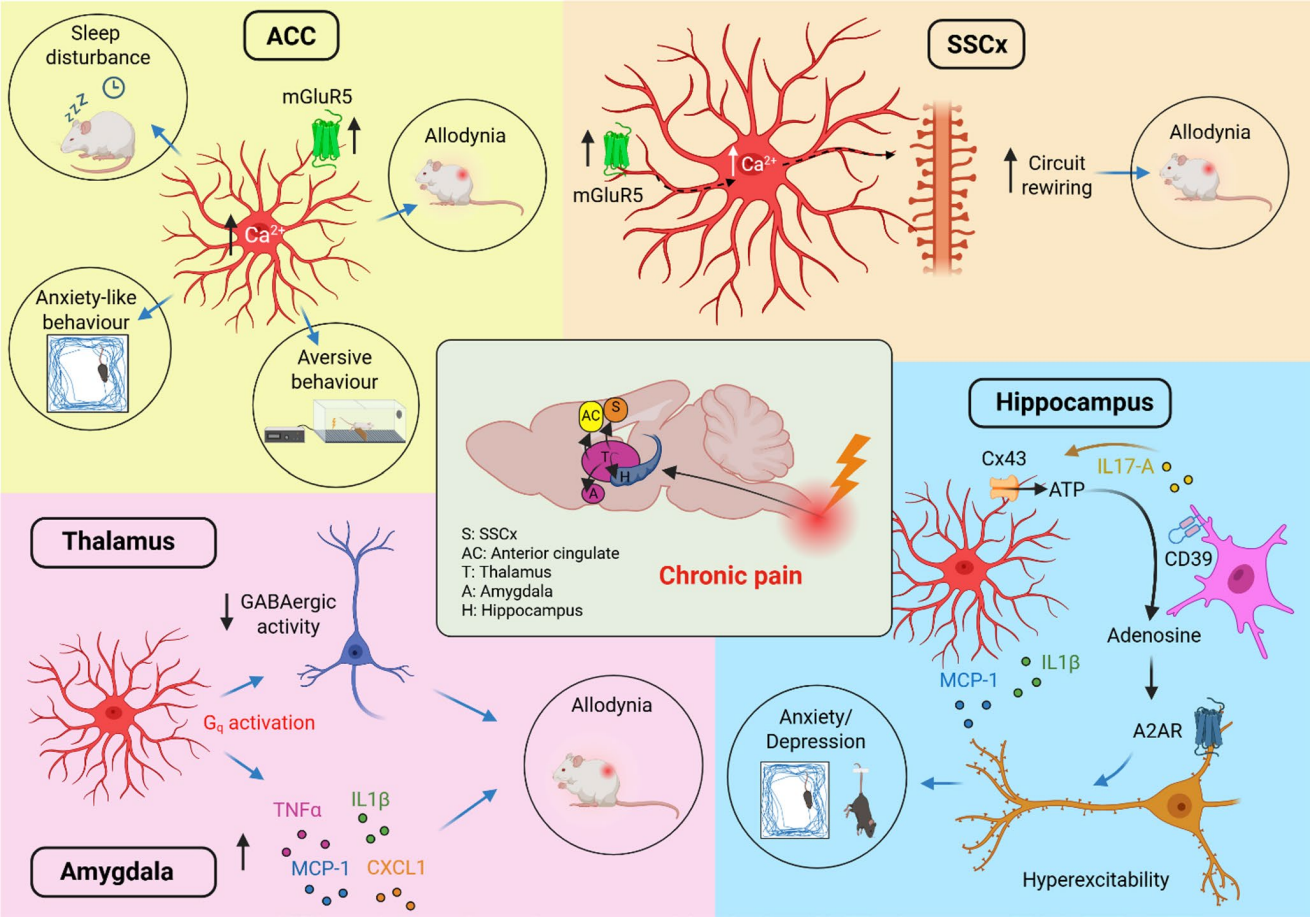
Created in BioRender. Chiavegato, A. (2025). https://BioRender.com/42hblyi

## Introduction

The International Association for the Study of Pain (IASP) defines **pain** as *an unpleasant sensory and emotional experience associated with*,* or resembling that associated with*,* actual or potential tissue damage* [1, 2]. Organisms have evolved this mechanism to ensure self-protection against potential dangerous situations. Indeed, pain confers a biological advantage by serving as a warning signal of an injury or medical condition, thus promoting the avoidance of further injuries and encouraging healing and recovery.

In the physiological pathway of nociception, a noxious stimulus is transduced into an electrical signal by peripheral nociceptors, primary afferents whose cell bodies are in the dorsal root ganglia and whose axons contact secondary order neurons in the spinal cord. The nociceptive signal follows then the ascending path across brainstem and is conveyed to the thalamus through the direct spinothalamic tract or the indirect reticulothalamic tract. From the thalamus, the signal is relayed to a number of higher brain centers that are key players in the modulation of sensory, emotional and cognitive dimensions of pain, such as somatosensory cortex (SSCx), anterior cingulate cortex (ACC), insular cortex, prefrontal cortex, amygdala and hippocampus (HIP). From these regions, modulatory signals are conveyed into a descending path encompassing brainstem regions such as the periaqueductal gray (PAG) and rostroventral medulla (RVM), acting as a natural pain relief system that modulates the transmission of pain signals.

Under certain conditions, the sensation of pain persists beyond the normal recovery period of an injury or illness, becoming pathological and chronic and developing into a disease itself. Constant or recurrent pain can also be linked to persistent inflammation such as in diabetes or arthritis, or arise from a disease or its treatment, such as in the case of cancer and chemotherapy. The official definition of IASP describes **chronic pain** as a *pain that persists or recurs for more than 3 months* [3]. This debilitating condition, highly diffuse worldwide [4–6], is characterized by spontaneous pain (perceived in the absence of external stimuli), allodynia (pain in the presence of non-painful stimuli) and hyperalgesia (enhanced sensitivity to painful stimuli). Among the different forms of chronic pain, the most prevalent is **neuropathic pain**, which is caused by damage to or dysfunction of the somatosensory nervous system [7].

The development of neuropathic pain involves the central sensitization of nociceptive circuitry through functional and anatomical remodelling of neural connections, leading to dysfunctional circuit activation also in the absence of a painful stimulus. One fundamental mechanism that underlies the transition from acute to chronic pain in the central nervous system is thus synaptic plasticity. In acute pain, synaptic changes such as increased neurotransmitter release and receptor sensitivity temporarily enhance pain perception, serving as an adaptive response to injury, but these mechanisms become maladaptive during pain chronicization [8], with maladaptive neuroplasticity potentially raising at different levels in pain circuit. Neuropathic pain is accompanied by different factors, such as neuroinflammation, changes in long-term potentiation (LTP), altered excitatory/inhibitory balance and reduced synaptic flexibility, which all contribute to persistent hyperexcitability and exaggerated responses to stimuli even in the absence of ongoing tissue damage [8–12]. Concurrent upregulation of proteins involved in the modulation of synaptic plasticity and LTP, e.g. NMDA receptors and neurotrophic factors such as BDNF, leads to enhanced excitatory transmission and increased neuronal excitability [13, 14]. Alongside this increase in excitatory transmission, chronic pain is also characterized by reduced GABAergic inhibition, which has been attributed to decreased gamma-aminobutyric acid (GABA) synthesis, impaired receptor function or altered interneuron activity [11, 15]. The loss of GABAergic tone not only amplifies pain signals but also contributes to the spread of pain beyond the original injury site. Over time, the central nervous system undergoes a shift in which plastic changes that are usually reversible become more stable and resistant to modulation. This loss of synaptic adaptability may contribute to establish pain circuits and impair the ability to return to a physiological state [8].

### Neuroglial Cells in Chronic Pain

Within the central nervous system (CNS), glial cells play fundamental roles in maintaining brain homeostasis and modulating brain function. We here focus on astrocytes, acknowledged partners of neurons in different brain circuits underpinning physiological processes, as well as in the development of neuropathological conditions [16–22]. Many studies have highlighted the role of astrocytes and microglia in the regulation of chronic pain within the spinal cord [23, 24]. Both cell types undergo molecular and morphological changes during the transition from acute to chronic pain, releasing cytokines and other molecules that promote the establishment of a pro-inflammatory milieu and ultimately affect neuronal activity. The analysis of the timecourse of glial cells recruitment reveals that microglia activation peaks and resolves much earlier than that of astrocytes during chronic pain development, revealing a closer temporal association between astrocyte activation and chronic pain induction and upkeep. For a comprehensive synopsis of the literature on the inflammatory pathways triggered in spinal astrocytes and microglia during chronicization of pain, we refer the readers to a number of previous publications [25–33]. While much of the research has focused on the spinal cord, fewer studies have explored how astrocytes in the brain respond or contribute to chronic pain. Nevertheless, emerging evidence suggests that these cells exhibit region-specific changes in various brain areas in different models of neuropathic pain. The aim of this review is to gather the most recent evidence on the ability of astrocytes to modulate chronic pain in higher brain regions, ranging from the thalamus to somatosensory cortex, up to anterior cingulate cortex and amygdala. In addition, we will add some examples of recent literature revealing how astrocyte modulation can influence mood disorders or cognitive outputs associated to chronic pain. In the graphical abstract, we summarize the main topics and evidence we will present in this review. Before delving into this, however, we will briefly describe key aspects of astrocyte excitability for a better understanding of the mechanisms and implications discussed throughout the review.

### Astrocyte Excitability

Cell excitability can be defined as the intrinsic ability of a cell to generate rapid changes in specific physiological parameters in response to external - or internal - stimuli. This ability relies on the presence of tightly controlled electrical and/or chemical gradients across cellular membranes, combined with the expression of ion channels and/or transporters that can be switched between OFF and ON permeability states to allow ion fluxes. While the term excitability commonly recalls electrically excitable neurons, voltage-gated channels and action potential firing, another universal form of cell excitability is enclosed in intracellular Ca^2+^ changes, which are often triggered by metabotropic (G-protein coupled) receptor activation and Ca^2+^ release from intracellular stores. Astrocytes are electrically non-excitable cells that rely on tightly regulated gradients of various ions, such as Ca^2+^, Na^2+^, Cl^−^ and H^+^, to sustain cell homeostasis [34–37]. While multiple ions with their associated signaling pathways contribute to astrocyte function [38], the majority of scientific research has historically focused on astrocyte excitability mediated by dynamic intracellular Ca^2+^ signaling. Accordingly, most studies cited in this review employed Ca^2+^ dynamics as a readout for astrocyte activity during physiological or pathological processes, as well as modulatory tools to promote or inhibit astrocyte intracellular Ca^2+^ signal. To aid in the comprehension of the experimental work presented throughout this review, we here provide a brief introduction to these topics.

Astrocytes express metabotropic receptors for many neurotransmitters and neuromodulators, such as glutamate, GABA, adenosine triphosphate (ATP), acetylcholine, norepinephrine, dopamine, endocannabinoids [17, 39–48]. These receptors are characterized by high affinity, slow desensitization and multi-step signal amplification, associated to intracellular pathways using Ca^2+^ or cAMP as second messengers. Following metabotropic agonist binding to Gq receptors, the activation of phospholipase C (PLC) leads to inositol triphosphate (InsP_3_) production and Ca^2+^ release along electrochemical gradient through InsP_3_ receptors at the endoplasmic reticulum membrane [48]. Intracellular Ca^2+^ signals in astrocytes are highly variegate in term of both spatial and temporal features, especially at the level of fine processes contacting synapses [49], and their analysis along astrocyte soma and subcellular compartments is a current challenge in neuroglial research. Notably, increases in the intracellular concentration of Ca^2+^ in astrocytes can lead to a regulated release of gliotransmitters (including glutamate, D-serine, ATP, GABA etc.), which have been shown to affect synaptic transmission, as well as to modulate circuits and behaviour [50, 51]. Monitoring Ca^2+^ signals in astrocytes is thus crucial to decode their involvement in brain circuit regulation.

Similarly crucial is the ability to modulate Ca^2+^ signals in astrocytes. Optogenetic and chemogenetic approaches have been developed to allow targetable expression of non-native ionic channels and metabotropic receptors in different cells, with the goal of controlling cell activation or inhibition. However, while neurons are easier to tune through spatially and temporally controlled application of depolarizing or hyperpolarizing stimuli, astrocyte manipulation is more complex. For example, optogenetic activation of ion fluxes across astrocyte membrane cannot mimic the variegate spatial and temporal dynamics of intracellular Ca^2+^ signals accompanying release from internal stores. Chemogenetic activation of DREADDs (Designer Receptors Exclusively Activated by Designer Drugs), conversely, is closer to physiological astrocyte activation since it triggers the intracellular cascade initiated by G-protein receptors. However, while Gq DREADDs are consistently reported to induce Ca^2+^ activity in stimulated astrocytes, it is more difficult to interpret data obtained with Gi DREADDs. Although these latter receptors are classically coupled to cAMP (cyclic adenosine monophosphate) pathway, they have been reported in different studies to result in Ca^2+^ increases [52] or in negative Ca^2+^ modulation [53]. Despite these limitations in determining the exact direction of astrocyte modulation, evidence of circuit and behavioural effects following astrocyte manipulation supports the proof of concept that astrocytes can play a significant role in regulating brain physiology and pathology.

Finally, it is noteworthy to clarify here the expression “*astrocyte activation*”, which is commonly used to define distinct and sometimes unrelated events. On one hand, the term *activation* can refer to the development of reactive astrogliosis, a state characterized by alterations in astrocyte gene expression, including up-regulation of glial fibrillary acidic protein (GFAP), cytokines and inflammatory mediators, as well as changes in their morphology and complexity, potentially resulting in atrophy or hypertrophy (for appropriate nomenclature, please refer to the consensus article [54]). On the other hand, *activation* can also refer to the initiation of intracellular signals in astrocytes, such as Ca^2+^ increases, commonly elicited by chemical, chemogenetic or optogenetic stimulation. Finally, we can also describe *activation* of Gi DREADDs in astrocytes, regardless of their ultimate stimulatory or inhibitory effect on Ca^2+^ signaling. We therefore encourage readers to adopt a critical perspective when interpreting the term “*astrocyte activation*”, as its meaning can vary substantially depending on the experimental context.

### Thalamus

The thalamus is the first supraspinal region to receive the nociceptive stimuli from the spinal cord and it relays them to the somatosensory cortex for further processing [55]. Neuroimaging study in patients suffering from chronic low back pain have showed an increased expression of translocator protein (TSPO), a marker of activated astrocytes and microglia, particularly in the thalamus and in the somatosensory cortex, highlighting the possible involvement of neuroglial cells in human chronic pain [56].

A role for the thalamic astrocytes in modulating neuropathic pain associated with nerve damage in diabetes mellitus has been recently described [57]. In a rat model of diabetic neuropathic pain, characterized by mechanical hindpaw allodynia, the authors observed various alterations in thalamic astrocytes. Specifically, astrocytes in the paraventricular thalamus (PVT) from diabetic rats exhibited increased expression of the astrogliosis marker GFAP, along with significant enlargement of soma size, increased number of branches and enhanced structural complexity. Chemogenetic activation of the Gq signaling pathway in PVT astrocytes of control rats was sufficient to induce morphological changes similar to those described in diabetic rats, along with mechanical hindpaw allodynia. Conversely, chemogenetic activation of the Gi signaling pathway in PVT astrocytes reversed the astrogliosis-related alterations and, most importantly, exerted an analgesic effect in diabetic rats with mechanical allodynia. Overall, these findings suggest a critical role for PVT astrocytes in mediating diabetic neuropathic pain. The authors also investigated the activity marker c-fos in PVT neurons and found a significant decrease of c-fos expression in rats with diabetic neuropathic pain. Remarkably, activation of Gi signaling in PVT astrocytes– which reduced allodynia– increased the number of c-fos positive GABAergic neurons in diabetic neuropathic pain rats. Consistently, chemogenetic activation of PVT GABAergic neurons relieved mechanical allodynia in diabetic rats, while their inhibition induced mechanical allodynia in control rats. Collectively, these results highlight the role of the PVT in diabetic neuropathic pain, with astrocytic Gq and GABAergic neuron activation exerting opposing effects on pain processing. In addition, these data suggest that astrocyte activation during the development of mechanical allodynia may promote neuropathic pain by inhibiting GABAergic neurons.

### Amygdala

The thalamus, besides relaying information to cortical areas, is also directly and indirectly connected to the amygdala [58], a region primarily involved in the emotional perception of pain but also in pain modulation [59, 60]. Beyond investigating the role of thalamic astrocytes in diabetic neuropathic pain rats, the group of Chang-Xi reported that astrocytes from the basolateral amygdala (BLA) also play a crucial role in the same model [61]. Similarly to what reported for the thalamus, GFAP expression was markedly increased in the BLA of diabetic rats and chemogenetic activation of Gi or Gq signaling in BLA astrocytes produced an analgesic effect in diabetic rats and an algesic effect in control rats, respectively. This dual effect was associated with bidirectional changes in BLA expression levels of inflammatory cytokines such as TNF-α, IL-1β, CXCL1 and MCP-1, which were reduced after astrocytic Gi activation in diabetic rats and increased after astrocytic Gq activation in control rats. The authors also explored the mechanism of action of koumine, a novel alkaloid analgesic that does not induce tolerance or dependence, and could therefore be safer than conventional analgesics such as gabapentin. Intragastric administration of koumine, which exerted an analgesic effect in diabetic neuropathic pain rats, was found to attenuate GFAP expression in BLA astrocytes and, consistently, to suppress the inflammatory response in the BLA. Collectively, these results highlight the involvement of BLA astrocytes in the pathophysiology of diabetic neuropathic pain.

### Hypothalamus

Paraventricular hypothalamus (PVH) is a supraspinal region centrally involved in perceiving and decoding somatic and visceral pain [62], as well as a potential center of sensitization in chronic pancreatitis due to its functional connection with the pancreas and its known role in visceral pain during pancreatic cancer [63]. Chronic upper abdominal pain is a common symptom in patients with chronic pancreatic inflammation. A recent study by Luo and colleagues investigated the role of hypothalamic astrocytes in this type of visceral pain. In a chronic pancreatitis model induced by intraperitoneal injection of caerulein, the authors confirmed persistent abdominal mechanical allodynia lasting up to 28 days. Interestingly, fiber photometry revealed astrocyte Ca^2+^ hyperactivity in response to abdominal stimulation in caerulein-injected mice, whereas sham mice showed no such activation. Elevated spontaneous Ca^2+^ activity was also observed in injected mice, compared to control mice. To investigate the role of astrocyte Ca^2+^ hyperactivity in abdominal allodynia, the authors used the Gi-coupled kappa-opioid receptor (KORD), activated by the ligand Salvinorin B. In brain slices from mice expressing KORD in PVH astrocytes, treatment with Salvinorin B reduced both basal Ca^2+^ levels and spontaneous Ca^2+^ elevations. Although in vivo suppression of astrocyte Ca²⁺ activity was not directly assessed, KORD activation successfully reduced abdominal mechanical allodynia in mice with chronic pancreatitis. In these mice, the authors also observed hyperactivity of PVH glutamatergic neurons and down-regulation of the astrocyte glutamate transporter GLT-1 (the rodent homolog of human EAAT2). Notably, activation of Gi signaling in astrocytes via KORD was sufficient to reverse both alterations, suggesting that the reduction of astrocyte Ca^2+^ signaling in mice with chronic pancreatitis exerts an analgesic effect by restoring GLT-1 expression levels and normalizing extracellular glutamate levels and neuronal excitability. Supporting this hypothesis, systemic administration of the GLT-1 agonist LDN-212,320 also ameliorated PVH glutamatergic neuron hyperactivity and abdominal pain in caerulein-injected mice. Although the precise mechanisms underlying astrocyte Ca²⁺ hyperactivity and GLT-1 dysfunction remain unexplored, these results highlight the crucial role of astrocytic GLT-1 in the PVN during chronic pancreatitis-associated allodynia.

### Primary Somatosensory Cortex

The primary somatosensory cortex (SSCx) receives and processes afferent thalamocortical (TC) inputs from the ventral posterolateral (VPL) nucleus of the thalamus. It serves as the principal region where peripheral signals are decoded and interpreted as noxious stimuli [64]. Due to the importance of this region in pain perception, several studies have investigated the involvement of the somatosensory cortex in the development of chronic pain. As already mentioned, neuroglial activation has been reported in the somatosensory cortex from patients with chronic low back pain [56].

Other studies conducted in animal models have further elucidated the role of astrocytes from the SSCx in chronic pain. Kim and colleagues, using partial sciatic nerve ligation (PSL) to model chronic pain in mice, demonstrated a transient increase in dendritic spine turnover in the SSCx during the first week following PSL. Notably, this narrow time window coincides with the onset of mechanical allodynia [10]. In a successive work, the same group revealed that astrocytes in the somatosensory cortex exhibit an increase in the frequency of basal intracellular Ca^2+^ transients, temporally correlated with both increased dendritic spine formation and allodynia development. Astrocyte Ca^2+^ signals were shown to be sufficient and necessary to enhance spine formation in somatosensory cortical dendrites, revealing the central astrocyte contribution to maladaptive synaptic remodelling. Indeed, photolysis of Ca^2+^ caged compounds to generate Ca^2+^ elevations in SSCx astrocytes increased dendritic spine turnover in control mice, while buffering astrocyte Ca^2+^ signals through sustained BAPTA-AM infusion in PSL-injured mice prevented spine turnover changes. The increase in astrocyte Ca^2+^ activity and the consequent synapse remodelling underlying the pain sensation was due to the upregulation of the metabotropic glutamate receptor-5 (mGluR5) receptors within the first week following the PSL. Notably, pharmacological blockade of mGluR5 during this period significantly suppressed mechanical allodynia for at least one month– as did BAPTA-mediated astrocyte Ca^2+^ buffering or the use of transgenic mice devoid of IP_3_ receptors in astrocytes (IP3RKO), characterized by reduced Ca^2+^ increases [65]. Years later, the same group further confirmed the central role of astrocytic mGluR5 for the development of chronic pain. After PSL, mGluR5 upregulation occurs in astrocytes in both the somatosensory cortex and the spinal cord. Danjo and colleagues, using a Cre-inducible mouse line, were able to knock out mGluR5 specifically in cortical astrocytes without affecting spinal astrocytes. In line with previous findings obtained with pharmacological mGluR5 inhibition, astrocyte-specific mGluR5 knockout animals did not develop allodynia following PSL. Moreover, deletion of mGluR5 reduced spontaneous and evoked Ca²⁺ activity of astrocytes in SSCx, reversed astrocyte-dependent synaptogenesis and prevented neuronal activity enhancement induced by PSL. These findings are particularly fascinating, since targeting a single astrocytic receptor in the SSCx is sufficient to completely prevent the development of neuropathic pain [66].

Following a similar line of research, Takeda and collegues investigated the effect of manipulating astrocytes *after* allodynia was already established. The rationale underlying this work resides in the hypothesis that while early astrocyte activation can induce maladaptive connections, a successive astrocyte reactivation may revert them by exploiting the same mechanism, i.e. by triggering spine turnover. Activation of astrocytes two weeks after PSL was combined with the transient blockade of noxious afferent input (using TTX or lidocaine therapy). Increasing astrocytic Ca^2+^ activity with low intensity transcranial direct current stimulation (tDCS) or via DREADDs approach significantly relieved allodynia-like behaviours in mice, with effects lasting up to two months. Mechanistically, astrocyte activation via Gq-coupled DREADDs was sufficient to significantly and specifically reduce the PSL-induced maladaptive synapses by increasing spine turnover [67]. Similar observations were made in another study using a chemogenetic approach to manipulate astrocytic Ca^2+^ activity. Gq-coupled stimulation of astrocytes in the somatosensory cortex of rats suppressed acid-induced thermal hyperalgesia, while a Gi-coupled approach blocked acupuncture-induced analgesia (electrical stimulation using acupuncture needle) [68].

Taken together, these studies suggest that astrocytes, and more specifically the intracellular Ca^2+^ activity of astrocytes in the SSCx are involved in the establishment of the maladaptive circuits underlying pain sensation in the neuropathic models. Interestingly, a second activation of astrocytes appears to contribute to the restoration of physiological conditions acting on spine turnover.

### Anterior Cingulate Cortex

The anterior cingulate cortex (ACC) is a limbic region involved in numerous emotional and cognitive processes. It also serves as the major hub for affective pain perception and associated behavioural responses, such as aversion, anxiety and memory formation [69, 70]. Cumulative evidence from animal models demonstrate that chronic pain and anxiety-like behaviours are associated with ACC neuronal hyperactivity [71, 72], while ACC neuronal response in the human brain during peripheral nociception correlates with the unpleasantness of pain [73, 74]. In this context, a critical role is highlighted for ACC astrocytes in the modulation of the emotional response to pain and in the induction of anxiety-like or aversive behaviours.

ACC hyperactivation is described by Wei and colleagues in a rat model of chronic inflammatory pain, where astrocyte activation - characterized by gliosis and neuroinflammation, with concurrent up-regulation of GFAP, TNFα, Il-6 and IL-1β - contributes to the comorbidity of chronic pain with anxiety. The authors demonstrated that interfering with astrocytic Ca^2+^ activity by a Gi-coupled DREADDs approach was sufficient to alleviate both allodynia and anxiety behaviour induced by Complete Freund’s adjuvant (CFA) injection in the hind paw of rats [75]. Similarly, in a mouse neuropathic pain model induced by nerve injury, ACC astrocytes displayed the ability to modulate anxiety-like behaviour, with their Gi-coupled chemogenetic activation reducing neuronal c-fos expression and ameliorating anxiety-like behaviours tested via open-field and elevated plus maze tests [76]. In this work, however, the possible effects of astrocyte modulation on allodynia were not assessed.

Other studies suggest an active role of astrocytes of the ACC in negative emotions underlying maladaptive behaviours under chronic pain condition. Interestingly, ACC astrocytes contribute to pain-related aversive behaviour in rats [77]. In particular, in a visceral chronic pain model, β2-adrenergic receptors (β2AR) expressed by astrocytes are activated by noradrenaline from locus ceruleus (LC) projections on ACC and are responsible of pain-evoked aversive behaviour. Indeed, while optogenetic activation of LC projections to ACC astrocytes promotes the consolidation and memory of pain-aversive learning, specific knockdown of astrocytic β2AR by miRNAi impairs animal performance in the conditioned place aversion test. In a different study on escape/avoidance behaviour in rats with induced allodynia, local injection of L-alpha-aminoadipate, a cytotoxin relatively specific for astrocytes, completely reverses the escape/avoidance behaviour in rats without affecting the CFA-induced mechanical allodynia [78], supporting distinct roles of ACC astrocytes in affective pain processing.

In a mouse model of neuropathic pain, Shen and collegues describe an abnormal Ca^2+^ activity of ACC astrocytes, caused by the up-regulation of astrocytic mGluR5 and associated with elevated extracellular glutamate concentration, enhanced synaptic transmission and allodynia. Selective attenuation of Gq signaling pathway through iβARK [79] or targeted RNA silencing of mGluR5 in astrocytes were each sufficient to restore normal astrocytic Ca^2+^ activity and significantly reduce both synaptic hypexcitability and mechanical allodynia. Additionally, Gq- coupled chemogenetic activation of ACC astrocytes exacerbated neuropathic pain compared to controls, although it was insufficient to induce pain by itself, suggesting a pain-facilitating role of these cells [80]. Interestingly, as already described in a previous section, comparable results on mGluR5 re-expression were observed in the somatosensory cortex. These findings suggest that mGluR5 plays a crucial role in modulating the transition from acute to chronic pain across different brain regions involved in pain processing, highlighting the mGluR5 pathway as a promising therapeutic target for reducing allodynia.

Notably, rodent models of sciatic nerve ligation or chronic inflammatory pain often show an imbalance between glutamate and GABA, with a shift toward elevated glutamate levels [71, 75, 81]. Yamashita and colleagues suggest a model in which the excess of glutamate induces Ca^2+^ dependent translocation of the GABA transporter 3 (GAT3) to the astrocyte membrane. Interestingly, optogenetic activation of ACC astrocytes led to increased wakefulness and reduced NREM sleep, mimicking conditions experienced by patients suffering sleep disorder in neuropathic pain [71].

### Hippocampus

The hippocampus plays a pivotal role in spatial memory and emotional regulation and it has been implicated in the development of mood disorders following nerve injury [82–84], as well as in comorbid cognitive impairments [85]. Notably, patients suffering from trigeminal neuralgia exhibit reduced hippocampal volume [86], and studies in rodent models of persistent pain have revealed multiple hippocampal abnormalities, including impaired function and altered neurogenesis [84, 87]. The research field on the contribution of hippocampus to pain modulation is relatively new. A recent work, for example, highlighted the direct participation of ventral hippocampus to the modulation of pain behaviours, through ensembles of nociceptive neurons showing inhibitory responses to noxious stimuli and encoding pain modality. Activation of the projectons of these neuronal ensembles to basolateral amygdala and infralimbic cortex is sufficient to mitigate pain behaviour [88].

To our knowledge, no study has yet addressed the possible role of astrocytes in hippocampal pathways contributing to chronic pain. We here report some examples of the involvement of astrocyte from ventral hippocampus in behavioral and mood alterations associated to neuropathic pain. The study of Fiore and Austin, for instance, disclosed an remarkable correlation between hippocampal neuroglia activation and exploratory behaviour of rats. The authors exposed rats to sciatic nerve chronic constriction injury (CCI) and observed different behavioural phenotypes. While all rats developed mechanical allodynia and impaired motor activity, only a subset of animals exhibited long-lasting deficits in exploratory behaviour. Interestingly, only this subgroup of rats exhibited astrocytic atrophy, with decreased GFAP expression, reduced IBA1 immunoreactivity, and elevated levels of pro-inflammatory mediators such as IL-1β and MCP-1, particularly in the ventral hippocampus [89]. While this work is more descriptive and correlative, another recent study used direct, functional activation of astrocytes from dorsal hippocampus via Gq DREADDs in a spared nerve injury (SNI) model. This paradigm induces neuropathic pain and cognitive dysfunction, associated with decreased levels of IL-33, a cytokine involved in synaptic plasticity and protection from oxidative stress. Astrocyte chemogenetic activation led to the recovery of IL-33 levels, reduced oxidative stress, restored synaptic connectivity and finally improved cognitive function [90]. In a model of clinical trigeminal neuropathic pain, chronic constriction of the infraorbital nerve (CION) induced allodynia and anxiety/depression-like behaviour, as well as gliosis in the CA1 region of the ventral hippocampus. Furthermore, CION triggered microglia activation, which in turn release IL-17 A to recruit astrocytes and induce a Ca²⁺-dependent ATP release from these cells. Notably, inhibiting astrocytic intracellular Ca^2+^ elevations, blocking connexin 43 hemichannels or preventing ATP degradation via the microglial CD39 enzyme were each sufficient to rescue the anxiety/depression-like behaviour. The authors ultimately propose a model in which anxiety/depression-like behaviour following CION depends on microglial activation inducing ATP release by astrocytes. This ATP is then degraded by microglial CD39 into adenosine, which increases the excitability of CA1 pyramidal neurons [91]. These findings support a deep connection between dysfunctional hippocampal glial communication and mood alterations in chronic pain.

## Conclusions and Future Directions

In conclusion, a substantial body of evidence highlights the central role of supraspinal astrocytes in the modulation of chronic pain across multiple brain regions involved in nociceptive processing. Several studies have shown that astrocytes are active players in different processes underlying chronic pain such as neuroinflammation, synaptic remodeling and regulation of excitatory/inhibitory balance. In particular, astrocytic intracellular Ca^2+^ dynamics and mGluR5-mediated signaling appear to be crucial for the transition from acute to chronic pain. Notably, astrocytes are not only involved in the establishment of allodynia, but they seem to play a critical role also in modulating the emotional and cognitive dimensions of chronic pain. While additional studies are necessary to fully recognize the role of astrocytes in brain regions involved in pain processing, the findings so far obtained by the scientific community encourage further research to pave the way for innovative therapeutic strategies targeting astrocyte-specific mechanisms, potentially offering more selective and long-lasting relief for neuropathic pain and its associated neuropsychiatric comorbidities.

## Data Availability

No datasets were generated or analysed during the current study.

## References

[1] Raja SN, Carr DB, Cohen M et al (2020) The revised international association for the study of pain definition of pain: concepts, challenges, and compromises. Pain 161:1976–1982. 10.1097/j.pain.0000000000001939 32694387 10.1097/j.pain.0000000000001939 PMC7680716

[2] IASP Subcommittee on Taxonomy (1979) Pain terms: a list with definitions and notes on usage. Recommended by the IASP subcommittee on taxonomy. Pain 6:249. https://www.iasp-pain.org/460932

[3] Treede R-D, Rief W, Barke A et al (2019) Chronic pain as a symptom or a disease: the IASP classification of chronic pain for the international classification of diseases (ICD-11). Pain 160:19–27. 10.1097/j.pain.0000000000001384 30586067 10.1097/j.pain.0000000000001384

[4] Zimmer Z, Fraser K, Grol-Prokopczyk H, Zajacova A (2022) A global study of pain prevalence across 52 countries: examining the role of country-level contextual factors. Pain 163:1740–1750. 10.1097/j.pain.0000000000002557 35027516 10.1097/j.pain.0000000000002557 PMC9198107

[5] Lucas JW, Sohi I (2024) Chronic pain and High-impact chronic pain in U.S. Adults, 2023. NCHS Data Brief CS355235. 10.15620/cdc/169630 10.15620/cdc/169630 PMC11726267 39751180

[6] Rometsch C, Martin A, Junne F, Cosci F (2025) Chronic pain in European adult populations: a systematic review of prevalence and associated clinical features. Pain 166:719–731. 10.1097/j.pain.0000000000003406 40101218 10.1097/j.pain.0000000000003406 PMC11921450

[7] van Hecke O, Austin SK, Khan RA et al (2014) Neuropathic pain in the general population: A systematic review of epidemiological studies. Pain 155:654–662. 10.1016/j.pain.2013.11.013 24291734 10.1016/j.pain.2013.11.013

[8] Song Q, Zhang ES, Liang Z Y (2024) Neuroplasticity in the transition from acute to chronic pain. Neurotherapeutics 21:e00464. 10.1016/j.neurot.2024.e00464 39438166 10.1016/j.neurot.2024.e00464 PMC11585895

[9] Cha MH, Kim DS, Cho ZH et al (2009) Modification of cortical excitability in neuropathic rats: A voltage-sensitive dye study. Neurosci Lett 464:117–121. 10.1016/j.neulet.2009.08.024 19682547 10.1016/j.neulet.2009.08.024

[10] Kim SK, Nabekura J (2011) Rapid synaptic remodeling in the adult somatosensory cortex following peripheral nerve injury and its association with neuropathic pain. J Neurosci 31:5477–5482. 10.1523/JNEUROSCI.0328-11.2011 21471384 10.1523/JNEUROSCI.0328-11.2011 PMC6622722

[11] Cichon J, Blanck TJJ, Gan WB, Yang G (2017) Activation of cortical somatostatin interneurons prevents the development of neuropathic pain. Nat Neurosci 20:1122–1132. 10.1038/nn.4595 28671692 10.1038/nn.4595 PMC5559271

[12] Ji R-R, Xu Z-Z, Gao Y-J (2014) Emerging targets in neuroinflammation-driven chronic pain. Nat Rev Drug Discov 13:533–548. 10.1038/nrd4334 24948120 10.1038/nrd4334 PMC4228377

[13] Xiong H-Y, Hendrix J, Schabrun S et al (2024) The role of the Brain-Derived neurotrophic factor in chronic pain: links to central sensitization and neuroinflammation. Biomolecules 14:71. 10.3390/biom14010071 38254671 10.3390/biom14010071 PMC10813479

[14] Li XH, Miao HH, Zhuo M (2019) NMDA receptor dependent Long-term potentiation in chronic pain. Neurochem Res 44:531–538. 10.1007/s11064-018-2614-8 30109556 10.1007/s11064-018-2614-8 PMC6420414

[15] Lorenzo L-E, Magnussen C, Bailey AL et al (2014) Spatial and Temporal pattern of changes in the number of GAD65-Immunoreactive inhibitory terminals in the rat superficial dorsal Horn following peripheral nerve injury. Mol Pain 10:57. 10.1186/1744-8069-10-57 25189404 10.1186/1744-8069-10-57 PMC4164746

[16] Lia A, Di Spiezio A, Vitalini L et al (2023) Ion Channels and Ionotropic Receptors in Astrocytes: Physiological Functions and Alterations in Alzheimer’s Disease and Glioblastoma. Life 13:2038. 10.3390/life13102038 10.3390/life13102038 PMC10608464 37895420

[17] Lia A, Sansevero G, Chiavegato A et al (2023) Rescue of astrocyte activity by the calcium sensor STIM1 restores long-term synaptic plasticity in female mice modelling alzheimer’s disease. Nat Commun 14:1590. 10.1038/s41467-023-37240-2 36949142 10.1038/s41467-023-37240-2 PMC10033875

[18] Gómez-Gonzalo M (2025) Astrocytes in rodent Anxiety-Related behavior: role of calcium and beyond. Int J Mol Sci 26:2774. 10.3390/ijms26062774 40141416 10.3390/ijms26062774 PMC11943343

[19] Lee H-G, Wheeler MA, Quintana FJ (2022) Function and therapeutic value of astrocytes in neurological diseases. Nat Rev Drug Discov 21:339–358. 10.1038/s41573-022-00390-x 35173313 10.1038/s41573-022-00390-x PMC9081171

[20] Kastanenka KV, Moreno-Bote R, De Pittà M et al (2020) A roadmap to integrate astrocytes into systems neuroscience. Glia 68:5–26. 10.1002/glia.23632 31058383 10.1002/glia.23632 PMC6832773

[21] Giusti V, Kaur G, Giusto E, Civiero L (2024) Brain clearance of protein aggregates: a close-up on astrocytes. Mol Neurodegener 19:5. 10.1186/s13024-024-00703-1 38229094 10.1186/s13024-024-00703-1 PMC10790381

[22] Kofuji P, Araque A (2021) Astrocytes and behavior. Annu Rev Neurosci 44:49–67. 10.1146/annurev-neuro-101920-112225 33406370 10.1146/annurev-neuro-101920-112225 PMC8257756

[23] Chen G, Park CK, Xie RG et al (2014) Connexin-43 induces chemokine release from spinal cord astrocytes to maintain late-phase neuropathic pain in mice. Brain 137:2193–2209. 10.1093/brain/awu140 24919967 10.1093/brain/awu140 PMC4107738

[24] Chen G, Zhang Y-Q, Qadri YJ et al (2018) Microglia in pain: detrimental and protective roles in pathogenesis and resolution of pain. Neuron 100:1292–1311. 10.1016/j.neuron.2018.11.009 30571942 10.1016/j.neuron.2018.11.009 PMC6312407

[25] Li T, Chen X, Zhang C et al (2019) An update on reactive astrocytes in chronic pain. J Neuroinflammation 16:140. 10.1186/s12974-019-1524-2 31288837 10.1186/s12974-019-1524-2 PMC6615111

[26] Lu H-J, Gao Y-J (2023) Astrocytes in chronic pain: cellular and molecular mechanisms. Neurosci Bull 39:425–439. 10.1007/s12264-022-00961-3 36376699 10.1007/s12264-022-00961-3 PMC10043112

[27] Zhang T, Zhang M, Cui S et al (2023) The core of maintaining neuropathic pain: crosstalk between glial cells and neurons (neural cell crosstalk at spinal cord). Brain Behav 13:e2868. 10.1002/brb3.2868 36602945 10.1002/brb3.2868 PMC9927860

[28] Ji R-R, Donnelly CR, Nedergaard M (2019) Astrocytes in chronic pain and itch. Nat Rev Neurosci 20:667–685. 10.1038/s41583-019-0218-1 31537912 10.1038/s41583-019-0218-1 PMC6874831

[29] Chen Y, Feng X, Cheung C-W, Liu JA (2022) Mode of action of astrocytes in pain: from the spinal cord to the brain. Prog Neurobiol 219:102365. 10.1016/j.pneurobio.2022.102365 36228888 10.1016/j.pneurobio.2022.102365

[30] Zhao H, Alam A, Chen Q et al (2017) The role of microglia in the pathobiology of neuropathic pain development: what do we know? Br J Anaesth 118:504–516. 10.1093/bja/aex006 28403399 10.1093/bja/aex006

[31] Donnelly CR, Andriessen AS, Chen G et al (2020) Central nervous system targets: glial cell mechanisms in chronic pain. Neurotherapeutics 17:846–860. 10.1007/s13311-020-00905-7 32820378 10.1007/s13311-020-00905-7 PMC7609632

[32] Gwak YS, Kang J, Unabia GC, Hulsebosch CE (2012) Spatial and Temporal activation of spinal glial cells: role of gliopathy in central neuropathic pain following spinal cord injury in rats. Exp Neurol 234:362–372. 10.1016/j.expneurol.2011.10.010 22036747 10.1016/j.expneurol.2011.10.010 PMC3303938

[33] Cheng T, Xu Z, Ma X (2022) The role of astrocytes in neuropathic pain. Front Mol Neurosci 15:1007889. 10.3389/fnmol.2022.1007889 36204142 10.3389/fnmol.2022.1007889 PMC9530148

[34] Bazargani N, Attwell D (2016) Astrocyte calcium signaling: the third wave. Nat Neurosci 19:182–189. 10.1038/NN.4201 26814587 10.1038/nn.4201

[35] Rose CR, Verkhratsky A (2024) Sodium homeostasis and signalling: the core and the hub of astrocyte function. Cell Calcium 117:102817. 10.1016/j.ceca.2023.102817 37979342 10.1016/j.ceca.2023.102817

[36] Untiet V (2024) Astrocytic chloride regulates brain function in health and disease. Cell Calcium 118:102855. 10.1016/J.CECA.2024.102855 38364706 10.1016/j.ceca.2024.102855

[37] Verkhratsky A, Untiet V, Rose CR (2020) Ionic signalling in astroglia beyond calcium. J Physiol 598:1655–1670. 10.1113/JP277478 30734296 10.1113/JP277478

[38] Verkhratsky A, Semyanov A, Zorec R (2020) Physiology of astroglial excitability. Function 1:zqaa016. 10.1093/function/zqaa016 35330636 10.1093/function/zqaa016 PMC8788756

[39] Pasti L, Volterra A, Pozzan T, Carmignoto G (1997) Intracellular calcium oscillations in astrocytes: A highly plastic, bidirectional form of communication between neurons and astrocytes in situ. J Neurosci 17:7817–7830. 10.1523/jneurosci.17-20-07817.1997 9315902 10.1523/JNEUROSCI.17-20-07817.1997 PMC6793927

[40] Requie LM, Gómez-Gonzalo M, Speggiorin M et al (2022) Astrocytes mediate long-lasting synaptic regulation of ventral tegmental area dopamine neurons. Nat Neurosci 25:1639–1650. 10.1038/s41593-022-01193-4 36396976 10.1038/s41593-022-01193-4

[41] Cahill MK, Collard M, Tse V et al (2024) Network-level encoding of local neurotransmitters in cortical astrocytes. Nature 629:146–153. 10.1038/s41586-024-07311-5 38632406 10.1038/s41586-024-07311-5 PMC11062919

[42] Lia A, Zonta M, Requie LM, Carmignoto G (2019) Dynamic interactions between GABAergic and astrocytic networks. Neurosci Lett 689:14–20. 10.1016/j.neulet.2018.06.026 29908949 10.1016/j.neulet.2018.06.026

[43] Henriques VJ, Chiavegato A, Carmignoto G, Gómez-Gonzalo M (2022) Astrocytes modulate somatostatin interneuron signaling in the visual cortex. Cells 11:1400. 10.3390/cells11091400 35563706 10.3390/cells11091400 PMC9102536

[44] Lohr C (2023) Role of P2Y receptors in astrocyte physiology and pathophysiology. Neuropharmacology 223:109311. 10.1016/j.neuropharm.2022.109311 36328064 10.1016/j.neuropharm.2022.109311

[45] Araque A, Martín ED, Perea G et al (2002) Synaptically released acetylcholine evokes Ca2 + Elevations in astrocytes in hippocampal slices. J Neurosci 22:2443–2450. 10.1523/jneurosci.22-07-02443.2002 11923408 10.1523/JNEUROSCI.22-07-02443.2002 PMC6758296

[46] Li WP, Su XH, Hu NY et al (2022) Astrocytes mediate cholinergic regulation of adult hippocampal neurogenesis and memory through M1 muscarinic receptor. Biol Psychiatry 92:984–998. 10.1016/j.biopsych.2022.04.019 35787318 10.1016/j.biopsych.2022.04.019

[47] Speggiorin M, Chiavegato A, Zonta M, Gómez-Gonzalo M (2024) Characterization of the astrocyte calcium response to norepinephrine in the ventral tegmental area. Cells 14:24. 10.3390/cells14010024 39791726 10.3390/cells14010024 PMC11720743

[48] Kofuji P, Araque A (2021) G-Protein-Coupled receptors in Astrocyte–Neuron communication. Neuroscience 456:71–84. 10.1016/j.neuroscience.2020.03.025 32224231 10.1016/j.neuroscience.2020.03.025 PMC8817509

[49] Lia A, Henriques VJ, Zonta M et al (2021) Calcium signals in astrocyte microdomains, a decade of great advances. Front Cell Neurosci 15:673433. 10.3389/fncel.2021.673433 34163329 10.3389/fncel.2021.673433 PMC8216559

[50] Kofuji P, Araque A (2021) Astrocytes and behavior. Annu Rev Neurosci 44:49. 10.1146/ANNUREV-NEURO-101920-112225 33406370 10.1146/annurev-neuro-101920-112225 PMC8257756

[51] Araque A, Carmignoto G, Haydon PG et al (2014) Gliotransmitters travel in time and space. Neuron 81:728–739. 10.1016/J.NEURON.2014.02.007 24559669 10.1016/j.neuron.2014.02.007 PMC4107238

[52] Durkee CA, Covelo A, Lines J et al (2019) G i/o protein-coupled receptors inhibit neurons but activate astrocytes and stimulate gliotransmission. Glia 67:1076–1093. 10.1002/glia.23589 30801845 10.1002/glia.23589 PMC6462242

[53] Luo R, Hu X, Li X et al (2024) Dysfunctional astrocyte glutamate uptake in the hypothalamic paraventricular nucleus contributes to visceral pain and anxiety-like behavior in mice with chronic pancreatitis. Glia 72:2022–2037. 10.1002/glia.24595 39046219 10.1002/glia.24595

[54] Escartin C, Galea E, Lakatos A et al (2021) Reactive astrocyte nomenclature, definitions, and future directions. Nat Neurosci 24:312–325. 10.1038/s41593-020-00783-4 33589835 10.1038/s41593-020-00783-4 PMC8007081

[55] Galdino G, Veras FP, dos Anjos-Garcia T (2024) The role of the thalamus in nociception: important but forgotten. Brain Sci 14(741). 10.3390/brainsci14080741 10.3390/brainsci14080741 PMC11352386 39199436

[56] Loggia ML, Chonde DB, Akeju O et al (2015) Evidence for brain glial activation in chronic pain patients. Brain 138:604–615. 10.1093/brain/awu377 25582579 10.1093/brain/awu377 PMC4339770

[57] Chen J, Yang L, Shen J et al (2025) Distinct roles of astrocytes and GABAergic neurons in the paraventricular thalamic nucleus in modulating diabetic neuropathic pain. J Neurosci 45:e1013242024. 10.1523/JNEUROSCI.1013-24.2024 39622642 10.1523/JNEUROSCI.1013-24.2024 PMC11841761

[58] Li D, Li Y-C, Zhu Z-Y et al (2025) The paraventricular thalamus mediates visceral pain and anxiety-like behaviors via two distinct pathways. Neuron 49:455–466. 10.1016/j.neuron.2025.04.019 10.1016/j.neuron.2025.04.019 40345185

[59] Cai YQ, Wang W, Paulucci-Holthauzen A, Pan ZZ (2018) Brain circuits mediating opposing effects on emotion and pain. J Neurosci 38:6340–6349. 10.1523/JNEUROSCI.2780-17.2018 29941444 10.1523/JNEUROSCI.2780-17.2018 PMC6041794

[60] Thompson JM, Neugebauer V (2017) Amygdala plasticity and pain. Pain Res Manag 2017:1–12. 10.1155/2017/8296501 10.1155/2017/8296501 PMC5742506 29302197

[61] Lu JS, Yang L, Chen J et al (2023) Basolateral amygdala astrocytes modulate diabetic neuropathic pain and May be a potential therapeutic target for Koumine. Br J Pharmacol 180:1408–1428. 10.1111/bph.16011 36519959 10.1111/bph.16011

[62] Zhang F-C, Li D (2024) Neuron distinct circuits and molecular targets of the paraventricular hypothalamus Decode visceral and somatic pain. Neuron 112:3734–3749e5. 10.1016/J.NEURON.2024.08.024 39326407 10.1016/j.neuron.2024.08.024

[63] Ji N-N, Cao S, Song X-L et al (2024) Glutamatergic neurons in the paraventricular nucleus of the hypothalamus participate in the regulation of visceral pain induced by pancreatic cancer in mice. Hepatobiliary Surg Nutr 13:258–272. 10.21037/hbsn-23-442 38617474 10.21037/hbsn-23-442 PMC11007342

[64] Ziegler K, Folkard R, Gonzalez AJ et al (2023) Primary somatosensory cortex bidirectionally modulates sensory gain and nociceptive behavior in a layer-specific manner. Nat Commun 14:1–18. 10.1038/s41467-023-38798-7 37225702 10.1038/s41467-023-38798-7 PMC10209111

[65] Kim SK, Hayashi H, Ishikawa T et al (2016) Cortical astrocytes rewire somatosensory cortical circuits for peripheral neuropathic pain. J Clin Invest 126:1983–1997. 10.1172/JCI82859 27064281 10.1172/JCI82859 PMC4855913

[66] Danjo Y, Shigetomi E, Hirayama YJ et al (2022) Transient astrocytic mGluR5 expression drives synaptic plasticity and subsequent chronic pain in mice. J Exp Med 219:e20210989. 10.1084/jem.20210989 35319723 10.1084/jem.20210989 PMC8952801

[67] Takeda I, Yoshihara K, Cheung DL et al (2022) Controlled activation of cortical astrocytes modulates neuropathic pain-like behaviour. Nat Commun 13:1–12. 10.1038/S41467-022-31773-8 35835747 10.1038/s41467-022-31773-8 PMC9283422

[68] Ye Q, Li J, Ren W-J et al (2024) Astrocyte activation in hindlimb somatosensory cortex contributes to electroacupuncture analgesia in acid-induced pain. Front Neurol 15:1348038. 10.3389/fneur.2024.1348038 38633538 10.3389/fneur.2024.1348038 PMC11021577

[69] Rolls ET (2019) The cingulate cortex and limbic systems for emotion, action, and memory. Brain Struct Funct 224:3001–3018. 10.1007/s00429-019-01945-2 31451898 10.1007/s00429-019-01945-2 PMC6875144

[70] Xiao X, Ding M, Zhang Y-Q (2021) Role of the anterior cingulate cortex in translational pain research. Neurosci Bull 37:405–422. 10.1007/s12264-020-00615-2 33566301 10.1007/s12264-020-00615-2 PMC7954910

[71] Yamashita A, Hamada A, Suhara Y et al (2014) Astrocytic activation in the anterior cingulate cortex is critical for sleep disorder under neuropathic pain. Synapse 68:235–247. 10.1002/syn.21733 24488840 10.1002/syn.21733

[72] Shao F-B, Fang J-F, Wang S et al (2021) Anxiolytic effect of GABAergic neurons in the anterior cingulate cortex in a rat model of chronic inflammatory pain. Mol Brain 14:139. 10.1186/s13041-021-00849-9 34507588 10.1186/s13041-021-00849-9 PMC8431944

[73] Rainville P, Duncan GH, Price DD et al (1997) Pain affect encoded in human anterior cingulate but not somatosensory cortex. Science 277:968–971. 10.1126/science.277.5328.968 9252330 10.1126/science.277.5328.968

[74] Kwan CL, Crawley AP, Mikulis DJ, Davis KD (2000) An fMRI study of the anterior cingulate cortex and surrounding medial wall activations evoked by noxious cutaneous heat and cold stimuli. Pain 85:359–374. 10.1016/S0304-3959(99)00287-0 10781909 10.1016/S0304-3959(99)00287-0

[75] Wei N, Guo Z, Qiu M et al (2024) Astrocyte activation in the ACC contributes to comorbid anxiety in chronic inflammatory pain and involves in the Excitation-Inhibition imbalance. Mol Neurobiol 61:6934–6949. 10.1007/s12035-024-04027-5 38363535 10.1007/s12035-024-04027-5

[76] Zhou Q, Zhong Q, Liu Z et al (2025) Modulating Anxiety-Like behaviors in neuropathic pain: role of anterior cingulate cortex astrocytes activation. CNS Neurosci Ther 31:e70227. 10.1111/cns.70227 39838823 10.1111/cns.70227 PMC11751476

[77] Iqbal Z, Lei Z, Ramkrishnan AS et al (2023) Adrenergic signalling to astrocytes in anterior cingulate cortex contributes to pain-related aversive memory in rats. Commun Biol 6:1–19. 10.1038/s42003-022-04405-6 36604595 10.1038/s42003-022-04405-6 PMC9816175

[78] Chen FL, Dong YL, Zhang ZJ et al (2012) Activation of astrocytes in the anterior cingulate cortex contributes to the affective component of pain in an inflammatory pain model. Brain Res Bull 87:60–66. 10.1016/j.brainresbull.2011.09.022 22004615 10.1016/j.brainresbull.2011.09.022

[79] Nagai J, Bellafard A, Qu Z et al (2021) Specific and behaviorally consequential astrocyte Gq GPCR signaling Attenuation in vivo with iβARK. Neuron 109:2256–2274e9. 10.1016/j.neuron.2021.05.023 34139149 10.1016/j.neuron.2021.05.023 PMC8418870

[80] Shen W, Chen F, Tang Y et al (2025) mGluR5-mediated astrocytes hyperactivity in the anterior cingulate cortex contributes to neuropathic pain in male mice. Commun Biol 8:266. 10.1038/s42003-025-07733-5 39979531 10.1038/s42003-025-07733-5 PMC11842833

[81] Niikura K, Furuya M, Narita M et al (2011) Enhancement of glutamatergic transmission in the cingulate cortex in response to mild noxious stimuli under a neuropathic pain-like state. Synapse 65:424–432. 10.1002/syn.20859 20812294 10.1002/syn.20859

[82] Meyer-Rosberg K, Kvarnström A, Kinnman E et al (2001) Peripheral neuropathic pain - A multidimensional burden for patients. Eur J Pain 5:379–389. 10.1053/eujp.2001.0259 11743704 10.1053/eujp.2001.0259

[83] Samwel HJA, Evers AWM, Crul BJP, Kraaimaat FW (2006) The role of helplessness, fear of pain, and passive pain-coping in chronic pain patients. Clin J Pain 22:245–251. 10.1097/01.ajp.0000173019.72365.f5 16514324 10.1097/01.ajp.0000173019.72365.f5

[84] Mokhtari T, Tu Y, Hu L (2019) Involvement of the hippocampus in chronic pain and depression. Brain Sci Adv 5:288–298. 10.26599/bsa.2019.9050025

[85] Zhao L, Zhang L, Tang Y, Tu Y (2025) Cognitive impairments in chronic pain: a brain aging framework. Trends Cogn Sci 29:570–585. 10.1016/j.tics.2024.12.004 39753445 10.1016/j.tics.2024.12.004

[86] Noorani A, Hung PSP, Zhang JY et al (2022) Pain relief reverses hippocampal abnormalities in trigeminal neuralgia. J Pain 23:141–155. 10.1016/j.jpain.2021.07.004 34380093 10.1016/j.jpain.2021.07.004

[87] Apkarian AV, Mutso AA, Centeno MV et al (2016) Role of adult hippocampal neurogenesis in persistent pain. Pain 157:418–428. 10.1097/j.pain.0000000000000332 26313405 10.1097/j.pain.0000000000000332 PMC4858177

[88] Shao S, Zheng Y, Fu Z et al (2023) Ventral hippocampal CA1 modulates pain behaviors in mice with peripheral inflammation. Cell Rep 42:112017. 10.1016/j.celrep.2023.112017 36662622 10.1016/j.celrep.2023.112017

[89] Fiore NT, Austin PJ (2018) Glial-cytokine-neuronal adaptations in the ventral Hippocampus of rats with affective behavioral changes following peripheral nerve injury. Neuroscience 390:119–140. 10.1016/j.neuroscience.2018.08.010 30125685 10.1016/j.neuroscience.2018.08.010

[90] Wang S, Yuan Y, Liu X et al (2025) IL-33 secreted from astrocytes alleviates cognitive impairment associated with neuropathic pain via oxidative stress in mice. J Neurophysiol 133:1919–1932. 10.1152/jn.00036.2025 40359065 10.1152/jn.00036.2025

[91] Lv XJ, Lv SS, Wang GH et al (2024) Glia-derived adenosine in the ventral hippocampus drives pain-related anxiodepression in a mouse model resembling trigeminal neuralgia. Brain Behav Immun 117:224–241. 10.1016/j.bbi.2024.01.012 38244946 10.1016/j.bbi.2024.01.012

